# Label-free 3D-CLEM Using Endogenous Tissue Landmarks

**DOI:** 10.1016/j.isci.2018.07.012

**Published:** 2018-07-20

**Authors:** Manja Luckner, Steffen Burgold, Severin Filser, Maximilian Scheungrab, Yilmaz Niyaz, Eric Hummel, Gerhard Wanner, Jochen Herms

**Affiliations:** 1 Department of Biology I, Biocenter Ludwig-Maximilians-University Munich, Planegg-Martinsried 82152, Germany; 2 German Center for Neurodegenerative Diseases (DZNE), Translational Brain Research, Munich 81377, Germany; 3 Center for Neuropathology, Ludwig-Maximilians-University Munich, Munich 81377, Germany; 4 Carl Zeiss Microscopy, Oberkochen 73447, Germany; 5 Munich Cluster of Systems Neurology (SyNergy), Munich 81377, Germany

**Keywords:** Neuroscience, Techniques in Neuroscience, Biological Sciences Research Methodologies, Biological Sciences Tools

## Abstract

Emerging 3D correlative light and electron microscopy approaches enable studying neuronal structure-function relations at unprecedented depth and precision. However, established protocols for the correlation of light and electron micrographs rely on the introduction of artificial fiducial markers, such as polymer beads or near-infrared brandings, which might obscure or even damage the structure under investigation. Here, we report a general applicable “flat embedding” preparation, enabling high-precision overlay of light and scanning electron micrographs, using exclusively endogenous landmarks in the brain: blood vessels, nuclei, and myelinated axons. Furthermore, we demonstrate feasibility of the workflow by combining *in vivo* 2-photon microscopy and focused ion beam scanning electron microscopy to dissect the role of astrocytic coverage in the persistence of dendritic spines.

## Highlights

• Flat embedding of vibratome sections for precise correlation of LM and SEM
• Endogenous landmarks are ideal fiducials for CLEM
• The glass slide serves as an absolute reference for precise alignment in yz
• Correlation of perisynaptic ensheathment and dendritic spine lifetime

## Graphical Abstract

**Figure:**
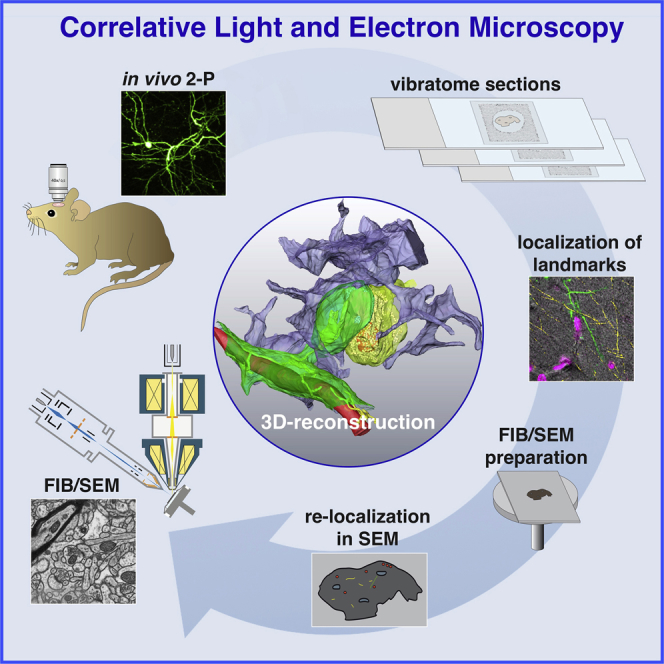

## Introduction

Studying biological key events within complex model systems relies on dynamic and functional imaging at optimum spatial and temporal resolution. Light microscopy (LM) allows visualization of dynamic cellular events in tissues, whereas electron microscopy (EM) remains the only method so far to reveal the complete subcellular architecture at nanometer resolution (Bourne and Harris, 2008). Correlative light and electron microscopy (CLEM) combines the advantages of both imaging modalities, allowing targeting the events of interest in space and time, using LM and subsequently resolving the ultrastructure of the same volume with EM (de Boer et al., 2015, Karreman et al., 2016a, Mironov and Beznoussenko, 2009). In particular, CLEM has greatly advanced our understanding of complex neuronal connectivity matrices by revealing the ultrastructural architecture and dynamics of neurites and synapses (Blazquez-Llorca et al., 2015, Genoud et al., 2006, Maco et al., 2013, Maco et al., 2014). However, a major methodological hurdle remains the correlation of LM and EM datasets by accurately tracking the position of the region of interest (ROI) within the EM specimen. For voluminous specimens such as the mouse brain, ROIs can be retrieved by screening serial thick (50–100 μm) vibratome sections of the tissue (Li et al., 2011). Nevertheless, serial EM imaging of large tissue samples is very cumbersome and results in unnecessarily large datasets. Currently, LM inspection to confine an ROI within the EM specimen is the most common approach for CLEM. For this purpose, fiducials are needed, which are detectable in both LM and EM. The ROIs can be marked by photo-oxidation of fluorophores (Grabenbauer et al., 2005), by affinity labeling with peroxidases (Knott et al., 2009) or by the use of exogenous fiducial markers like polymer beads (Kukulski et al., 2012), quantum dots (Masich et al., 2006), or near-infrared branding (NIRB) (Bishop et al., 2011). Although useful, these approaches require processing of the tissue samples and thereby might obscure the target structure or even deteriorate their ultrastructure. Alternatively, endogenous landmarks, which surround the ROI providing both LM and EM contrast, can be used as a guide to retrace the position of the ROI following EM processing (Karreman et al., 2016b). Unfortunately, resin embedding for EM preparations covers endogenous landmarks, thus prohibiting marker identification by EM. Although there are some protocols to reduce resin embedding, these methods comprise several delicate preparation steps or specialized equipment (Kizilyaprak et al., 2014, Belu et al., 2016, Lucas et al., 2017, Schieber et al., 2017). These issues were addressed by developing a “flat embedding” preparation to enable direct LM visualization of endogenous fiducial markers, present throughout the brain parenchyma. We show that blood vessels, nuclei, and myelinated axons can be used for precise correlation of LM and EM images with micrometer accuracy, allowing retrieval of structures as small as single synapses. A wide range of optical microscopic modalities, including wide-field, differential interference contrast (DIC), and confocal and reflectance microscopies can be used to visualize these endogenous landmarks with minimal labeling effort or even in a completely label-free manner. The feasibility of the protocol was confirmed by revealing the intimate interplay of perisynaptic astrocytic processes and dendritic spines, previously imaged by *in vivo* two-photon microscopy and subsequently relocated and imaged by focused ion beam scanning electron microscopy (FIB/SEM) with nanometer resolution.

## Results

### Natural Landmarks for 3D-CLEM

**Figure 1:**
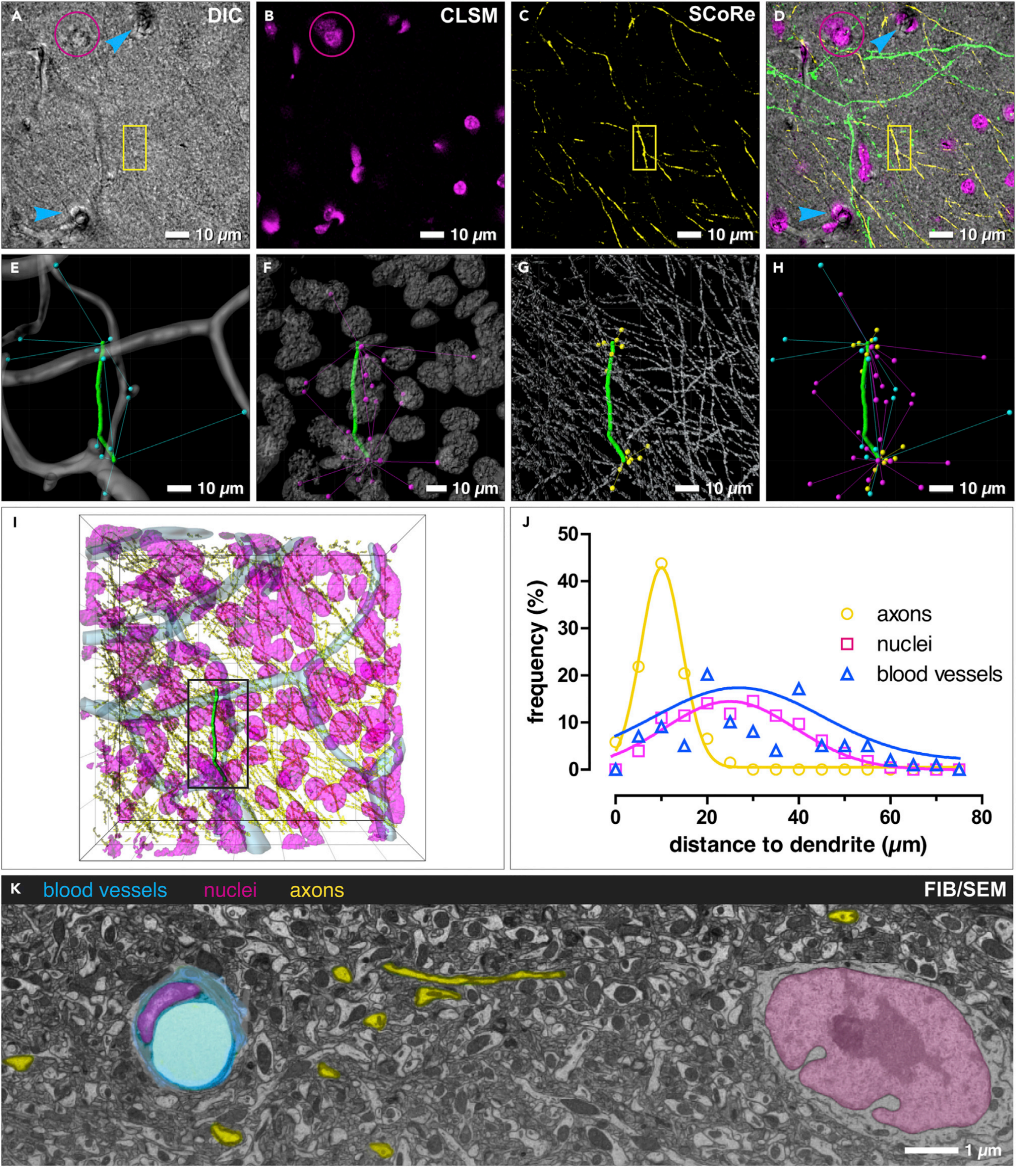
Availability and Precision of Endogenous CLEM Landmarks (A–C) Blood vessels (arrowheads), nuclei (magenta) and myelinated axons (yellow; boxed area) can be visualized by DIC (A), CLSM (B) and SCoRe (C) microscopy. (D) Overlay of DIC, CLSM, and SCoRe images. Blood vessels (arrowheads); nuclei (magenta); myelinated axons (yellow; boxed area). (E–H) 3D reconstructions of blood vessels (E), nuclei (F), and myelinated axons (G) with distance traces between target dendrite and closest landmarks (H). (I) Overlay of the 3D reconstructions of landmarks and the target dendrite. (J) Frequency distribution of correlative landmarks, plotted against their respective distance to the target dendrite. (K) FIB/SEM micrograph of cortical mouse brain tissue clearly represents blood vessels (blue), nuclei (magenta), and myelinated axons (yellow) by their typical shape and contrast. Related to Figures S1–S3.

Blood vessels, nuclei, and myelinated axons are excellent fiducial markers for 3D-CLEM since they fulfill the following criteria: (1) sufficient contrast in LM and EM, (2) distinctive size and shape, and (3) sufficient density to restrict the volume of correlation (Figure 1). They can be readily recognized by DIC (Figure 1A) and FIB/SEM (Figure 1K). The precise 3D position of these landmarks in proximity to the target dendrite can be mapped by various confocal microscopic techniques (Figures 1A–1C). Confocal laser scanning microscopy (CLSM) enables high-resolution imaging of nuclei, stained with the cell-permeant DNA-binding dye DRAQ5 (Figure 1B). Since DRAQ5 is a vital dye, tissue permeabilization can be omitted, which bears the risk of ultrastructural deterioration. In addition, spectral confocal reflectance microscopy (SCoRe) (Schain et al., 2014) can be used for direct, label-free visualization of myelinated axons (Figure 1C). Spatial distances between a randomly selected dendritic segment and its surrounding landmarks in cortical layer I were determined to confirm that landmarks are sufficient for precise CLEM alignment (Figures 1E–1J). The average distance between a dendrite and either blood vessels (Figure 1E) or nuclei (Figure 1F) equaled 25.5 μm or 26.7 μm, respectively (Figure 1J). The distinct morphology of both markers facilitated the identification of ROIs by triangulation of the landmarks. Myelinated axons (Figures 1C and 1G) are present in higher density and subsequently at closer vicinity to the target dendrite, with an average distance of 9 μm (Figure 1J), thus further increasing the precision of ROI retrieval.

### “Flat-Embedding” Preparation for CLEM

**Figure 2:**
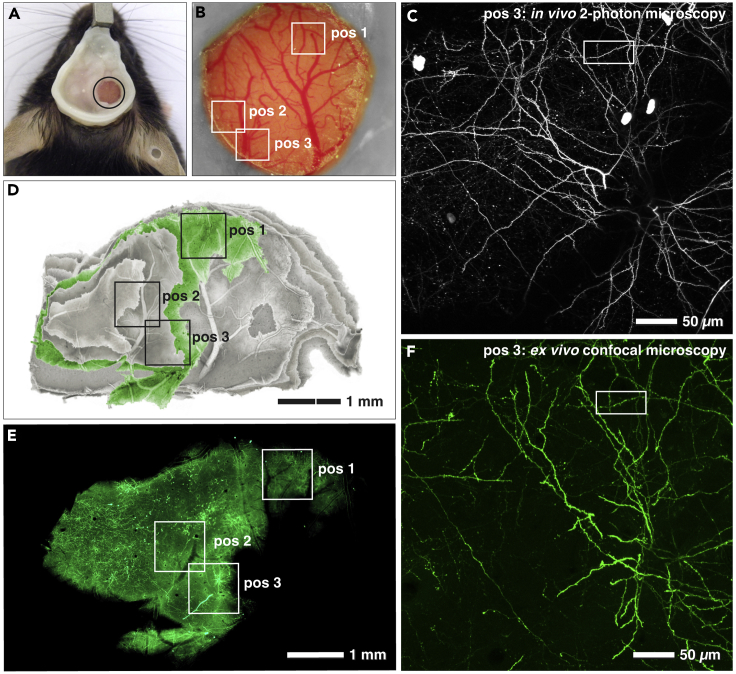
*In vivo* and *Ex Vivo* Light Microscopy for CLEM (A) Cranial window implantation gives optical access to the cortex of the mouse brain. (B) Magnified image section of cranial window in (A). The blood vessel pattern enables the retrieval of previously imaged positions (framed areas: pos 1–pos 3). (C) Maximum intensity projection of *in vivo* 2-photon image stack of pos 3 (framed in B). Framed area designates the target dendrite at the last imaging time point (day 41). (D and E) Reconstruction of the cortex by alignment of vibratome sections (D), based on the blood vessel pattern, facilitates identification of the brain slice (green) containing the target dendrites of three different positions (pos 1– pos 3) (E). (F) Maximum intensity projection of *ex vivo* CLSM image stack of pos 3 (D and E). Framed area designates the target dendrite [compare (F) with (C)].

To demonstrate the applicability and precision of the presented CLEM preparation method, several ROIs with Thy1.2-eGFP-expressing dendritic tufts in the somatosensory cortex of Thy1.2-GFP-M mice (Figures 2A–2C) were imaged by *in vivo* 2-photon microscopy and subsequently relocated in vibratome sections (Figures 2D–2F). Vibratome sections were immobilized on glass slides to maintain specimen orientation during LM and EM preparation. To identify the sections containing the ROI, brain slices were recorded with an epifluorescence microscope to reconstruct the dissected cortex with an imaging software (Adobe Photoshop), based on its unique blood vessel pattern (Figure 2D).

**Figure 3:**
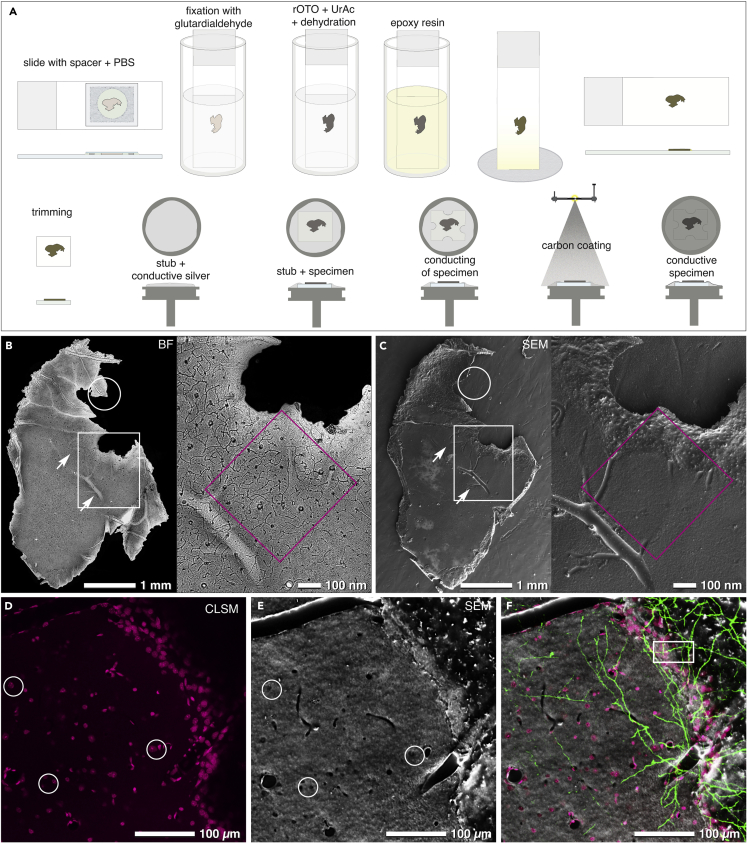
Sample Preparation and Retrieval of Landmarks (A) Vibratome sections are mounted onto a glass slide with a spacer and sealed by a coverslip. After LM, the coverslip and the spacer are removed. Post-fixation (glutardialdehyde, reduced osmium-ferrocyanide-thiocarbohydrazide-osmium (rOTO), uranyl acetate [UrAC]), dehydration and infiltration with epoxy resin are performed in vials. After removal of excess resin and polymerization, the slide is trimmed to appropriate size. The specimen is mounted with colloidal silver onto an aluminum stub, conducted with bridges of colloidal silver, and coated with carbon (15–20 nm) by evaporation. (B and C) Comparison of a bright field (BF) micrograph (B) with a scanning electron micrograph (C) of the selected vibratome slice (Figures 2 D and 2E). At low magnification, changes in morphology are easily recognized (B, C, circles). Intersected blood vessels are visible in both images (arrows), serving as the most prominent landmarks. (D–F) Optical sections of surface near nuclei (D; white circles) can be correlated to intersected nuclei, visible in SEM at higher voltages (20 kV) due to both topographic and material contrast (E; white circles). Superimposition of both signals (DRAQ5: magenta, nuclei; eGFP: green, dendrites) with the SEM image (F) serves as a precise map for localizing the target dendrite in top view (boxed area). Related to Figures S1–S3.

ROI-containing brain sections were further processed for EM (Figure 3A). Excessive resin, covering the brain tissue, was removed by draining and centrifugation to enable direct macroscopic inspection of the tissue surface (Figures 3B and 3C). Superimposition of bright field (Figure 3B) and SEM (Figure 3C) micrographs, based on the characteristic outline of the brain section, was sufficient for an immediate macroscopic correlation. The preparation protocol preserved the integrity of the specimens without major tissue shrinkage or corrugations (Figures 3B and 3C), as could be expected from dehydration. Suitable fiducials for CLEM could be identified in SEM due to the carbon coating of the specimen: since carbon has lower yields of backscattered electrons (BSE) and secondary electrons (SEs), compared with heavy metals, it appears transparent and superficial structures of the tissue become detectable. Structures lying in greater depth become visible by slightly increasing the accelerating voltage (Figure S1). The distinctive sizes and shapes of the apparent natural landmarks (blood vessels appear as channels or large holes, nuclei as dark dots) were used for the subsequent superimposition of light and electron micrographs, to define the target area in x/y direction (Figures 3D–3F).

### 3D Landmark Correlation of LM and FIB-SEM Datasets

**Figure 4:**
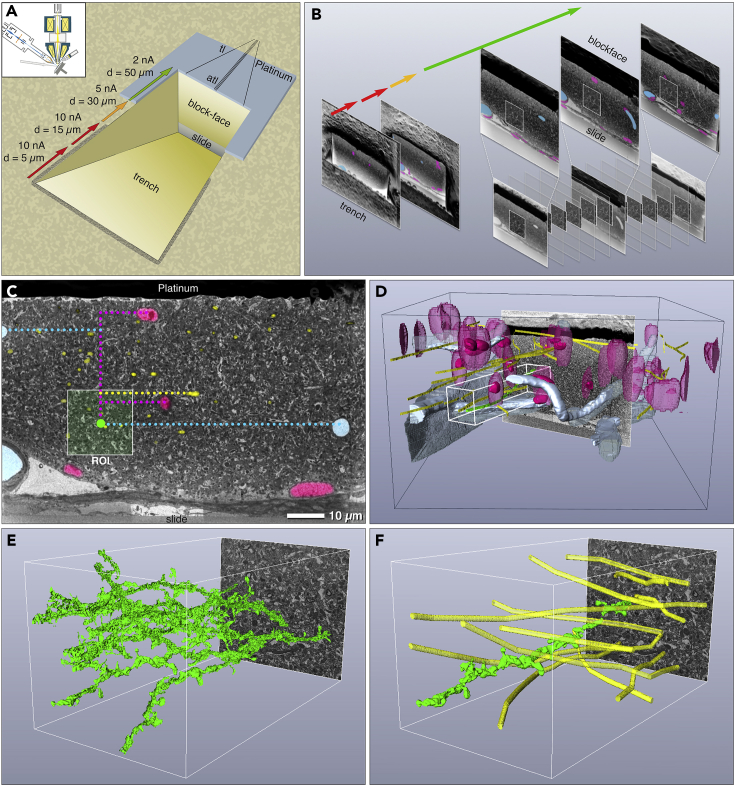
3D alignment of LM and FIB/SEM Tomograms (A) Economic trench milling in several steps: successive decrease in ion beam energy with increasing milling depth. FIB/SEM tomography is performed by eucentric tilting of the specimen to 54° into the coincidence point (inset). The target area is coated with approximately 1 μm platinum by ion beam deposition. Thin tracking lines (tl) and autotune lines (atl) serve for controlling the milling/imaging process (section thickness, focus, astigmatism). (B) High-resolution images (white squares) are taken every 15 nm of milling. In addition, key frames are taken in intervals of 1 μm in z-direction, providing micrographs for fast, “on the fly” 3D correlation of natural landmarks: blood vessels (blue), nuclei (magenta), and myelinated axons (yellow). (C) When reaching the final block face the region of interest (ROI; white square) with the target dendrite (green spot) is defined in x/y using the coordinates of the landmarks derived from the 3D LM data. Blood vessels (blue); nuclei (magenta); myelinated axons (yellow). (D) Superimposition of landmarks (blood vessels, nuclei, and axons; transparent) of the LM reconstructions (black box) with the FIB/SEM reconstructions of the corresponding structures (solid). (E) Preliminary fast reconstructions of several potential dendrites (green) in the target volume (white box) by an automatic labeling algorithm (*Magic Wand*, Amira™). (F) Usage of myelinated axons (yellow) as correlative marker to identify the target dendrite (green). Related to Video S1.

After identification of the target area on the specimen surface, FIB milling was used to gain access into the brain tissue containing the dendrite of interest, which was previously imaged by *in vivo* 2-photon microscopy. Milling time, and consequently costs, were reduced by milling the trench toward the volume of interest with high beam current, which was stepwise decreased, while approaching the final block face of 70 × 50 μm (Figures 4A–4C). Since block-face micrographs exhibit sufficient landmarks for correlation, the position of the ROI could be identified by correlating the FIB/SEM 3D reconstructions of nuclei and blood vessels with the corresponding CLSM 3D data in Amira (Figure 4D). A rough 3D reconstruction of the dense axonal network further facilitated the identification of the target dendrite (Figures 3E and 3F; Video S1), thus reducing the ROI to 15 × 15 μm (Figure 4C). The final ROI images were acquired with high resolution (pixel size in x/y: 5 nm) every 15 nm, whereas low-resolution overview images (pixel size in x/y: 27 nm) of the entire block-face area were recorded every 1 μm in z (Figures 4B and 4C) to confirm or eventually adjust the position of the ROI “on the fly.”

Video S1. 3D-Reconstruction of the Axonal Network Facilitates Identification of Target Dendrite, Related to Figure 4

### CLEM of Dendritic Spine Lifetime and Astrocytic Synapse Coverage

**Figure 5:**
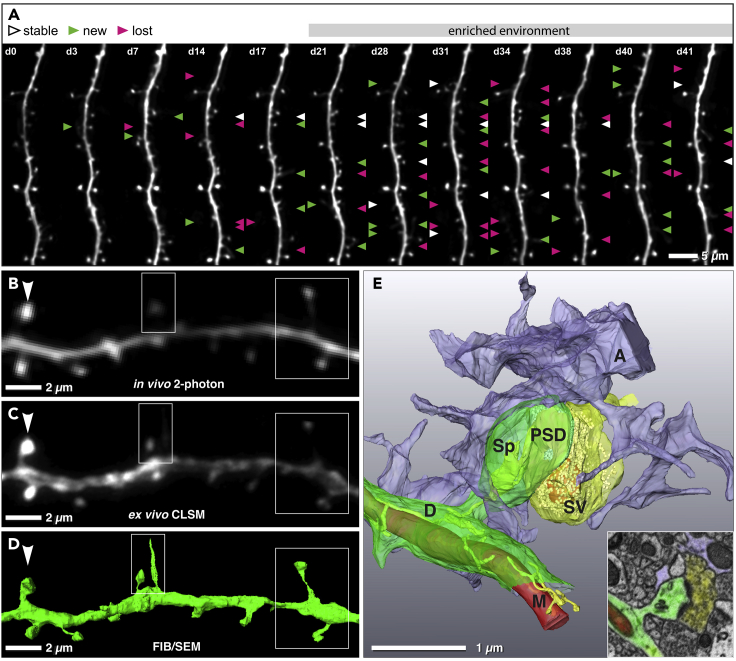
3D-CLEM of Cortical Tripartite Synapses (A) *In vivo* 2-photon micrographs of eGFP-labeled apical dendrites of layer V pyramidal neurons in the somatosensory cortex imaged before and during enriched environment. Enriched environment exposure started at day 18 and was continued until end of the imaging period. White arrowheads mark spines that formed newly and remained stable for at least two consecutive imaging time points; gained and lost spines are labeled with green and magenta arrowheads, respectively. Scale bar = 5 μm. (B–D) Comparative juxtaposition of the same dendritic segment recorded by *in vivo* 2-photon (B), *ex vivo* CLSM (C) and FIB/SEM microscopy (D). White boxes indicate dendritic spines that were detected in high-resolution CLSM microscopy and FIB/SEM; white arrowheads indicate spines that were detected in all imaging modalities. Scale bar = 2 μm. (E) 3D reconstructed FIB/SEM tomogram of a complete tripartite synapse (A, astrocyte; D, dendrite; M, mitochondrion; PSD, postsynaptic density; Sp, spine; SV, synaptic vesicles). Inset shows a single micrograph of the corresponding FIBS/SEM stack depicting a dendritic spine (green) with associated presynapse (yellow) and astrocyte (purple). Scale bar = 1 μm. Related to Figure S4.

The concept of the “tripartite synapse” refers to the functional integration and physical proximity of astrocytic processes with pre- and postsynaptic elements of the chemical synapse (Araque et al., 1999). However, experimental dissection of the morpho-functional relationship between these structures is hampered by the very small size of perisynaptic astrocytic processes (PAPs), which is below the resolution limit of conventional LM (Heller and Rusakov, 2015, Panatier et al., 2014). CLEM in combination with the “flat-embedding” protocol is suitable to study the morpho-functional interactions between PAPs and their corresponding synapses at an ultrastructural level. Hereby, we were able to investigate whether the extent of synaptic PAP coverage correlates with the lifetime of post-synaptic partners. To assess the lifetime of dendritic spines, chronic *in vivo* 2-photon microscopy in the somatosensory cortex of adult Thy1.2-GFP-M mice (Feng et al., 2000) was performed. The dynamics of eGFP-labeled spines on apical dendritic tufts of layer V pyramidal neurons were monitored before and during enriched environment exposure of mice (Jung and Herms, 2014). As documented by the micrograph time series (Figure 5A), enriched environment substantially and persistently promoted spinogenesis and thus increased the amount of newly formed spines accessible for lifetime analysis. Subsequently, target dendrites were relocated in vibratome sections and respective EM specimen by flat embedding and triangulation of natural landmarks. Correlation of *in vivo* 2-photon microscopy, *ex vivo* CLSM, and FIB/SEM 3D datasets (Figures 5A–5D) demonstrates that structural integrity of the corresponding dendrites and dendritic spines (Figures 2C–2F) was well preserved throughout CLEM preparation. Based on the FIB/SEM tomograms, tripartite synapses were reconstructed in 3D to determine the perimeter of the synaptic cleft and its astrocytic coverage (Figure 5E and Videos S2 and S3). Correlation of these structural parameters with dendritic spine lifetime revealed that the fraction of synaptic perimeter surrounded by PAPs on average amounts to 37% and scales neither with synaptic cleft area nor with spine age (Figures S4A and S4B). The synaptic perimeter of a few old spines (lifetime ≥41 days) was even completely devoid of PAPs.

Video S2. FIB/SEM 3D-Reconstruction of a Single Dendritic Element, Related to Figure 5

Video S3. High-Resolution FIB/SEM 3D-Reconstruction of a Single Tripartite Synapse, Related to Figure 5

## Discussion

Methodological hurdles in CLEM are the re-localization of a rather small target volume within a large tissue volume, changing sample orientation during the transition between different microscopy modalities, and structural distortions or preparation artifacts caused by artificial fiducials (de Boer et al., 2015, Grabenbauer, 2012, Karreman et al., 2016a). Current CLEM approaches, based on artificial fiducials, suffer from several drawbacks (Table 1): (1) labeling with electron-dense precipitate or fluorescent beads/quantum dots can obscure ultrastructural details; (2) fixation and harsh permeabilization conditions, required for antibody labeling, compromise ultrastructure; (3) delivery of tracers to living tissue may induce toxic side effect; and (4) NIRB is mainly used to mark the surface of the sample, as light scattering within the tissue limits the depth of NIRB (Karreman et al., 2014).

**Table 1:** Comparison of Common CLEM Protocols

	Pro	Con
“Flat embedding”	Fast Cheap Nondisruptive Suitable for all kind of tissues	Limited in vibratome section thickness of 50 μm
NIRB (Bishop et al., 2011)	Fast Suitable for all kind of tissues	Disruptive Fs-laser needed
DAB (Sonomura et al., 2013)	Direct visualization of the target structure	Masking ultrastructure
QDs (Masich et al., 2006)	Suitable for all kind of tissues	Masking ultrastructure Limited tissue penetration

NIRB, near-infrared branding; DAB, diaminobenzidine; QDs, quantum dots

To circumvent these technical drawbacks we introduce a “flat-embedding” protocol of vibratome sections, ideal for both SEM and FIB/SEM investigations (Figures 1, 2, S1, S2, and S3). Due to their thickness of approximately 50 μm, vibratome sections can be: (1) adequately fixed, even as large slices (e.g., 20 mm^2^), (2) investigated entirely at low and high magnifications with LM and SEM (Figures 3B and 3C), and (3) milled by FIB/SEM in their entire thickness (Figure 4C). Since tissue sections can be permanently immobilized on glass slides, their orientation does not change in the transition from LM to FIB/SEM (Figures 3B and 3C). The complex correlation of LM and SEM data can be achieved by a simple overlay of the LM image, depicting the sample surface, onto the SEM image of the resin-embedded section (Figure 3F), with its characteristic topography (SE image) and material contrast information (BSE image). In addition to the direct surface topography, subsurface information can be gathered at high voltages, as the BSE signal can be detected within a depth of approximately 3 μm at 25 kV (Figures S2 and S3). Thereby, the surface of the specimen becomes transparent and prominent structural features as nuclei and axons become visible with strong contrast (Figure S3). DIC microscopy yields sufficient resolution and depth of field to visualize blood vessels, nuclei, and myelinated axons simultaneously (Figure 1A). Furthermore, SCoRe microscopy can be applied to visualize myelinated axons based on their high refractive index in a label-free manner (Schain et al., 2014) (Figure 1C). Axons are excellent high-resolution fiducials for brain tissue, due to their high density and strong BSE signal in SEM (Figures 1K and S2). As nuclei and blood vessels are abundantly present in all animal tissues, this method can also be used for various organs like kidney, liver, and skin.

A reduction of the laborious FIB/SEM trench milling could be achieved by stepwise adjusting the ion beam current depending on the trench position, thus saving time and costs (Figures 4A and 4B). Monitoring the constantly increasing block face every 0.5–1 μm provides essential information about the position of the relevant landmarks, which can be reconstructed in 3D and compared with the LM stacks for possible corrections or fine adjustments (Figures 4C and 4D). Since the cross-section of the glass slide serves as absolute reference for precise alignment, an exact correlation is ensured (Figure 4C) (Luckner and Wanner, 2018). These improvements reduce the CLEM workflow and make artificial fiducials and delicate trimming needless (Kolotuev et al., 2012).

The high precision of the presented CLEM method was demonstrated by *in vivo* 2-photon microscopy of single dendritic spines and their subsequent identification within a resin-embedded tissue by FIB/SEM Detailed information could be gathered about dendritic spine lifetime and morphometric measurements of the corresponding tripartite synapse at nanometer resolution (Figure 5). Our data show that astrocytic coverage of the synaptic cleft does not proportionally scale with either the synapse size or the synapse age (Witcher et al., 2007), indicating that smaller, newly formed spines, as well as established larger spines, have equal access to extracellular glutamate, which is restricted by astroglial ensheathment of the synaptic perimeter. Thus, astrocytes may facilitate integration of synapses by preventing transmitter spillover between neighboring excitatory synapses (Ostroff et al., 2014). Although we did not detect a change of astrocytic coverage during synapse maturation under basal conditions, it has been shown to increase during periods of enhanced neuronal activity to augment glutamate clearance and preserve synaptic response (Genoud et al., 2006). Furthermore, the observation that the synaptic perimeters of some persistent spines are completely devoid of astroglial processes indicates that permanent astrocytic coverage might not be mandatory for the maintenance of excitatory synapse stability (Bernardinelli et al., 2014) (Figure S4).

Summarizing, we introduce a precise and efficient CLEM preparation method, which (1) circumvents the need of artificial fiducials, (2) is compatible with widely accessible optical microscopic techniques, and (3) is suitable for various scientific questions.

## Methods

All methods can be found in the accompanying Transparent Methods supplemental file.

## Supplemental Information

Document S1. Transparent Methods and Figures S1–S4

## References

[bib1] Araque A., Parpura V., Sanzgiri R.P., Haydon P.G. (1999). Tripartite synapses: glia, the unacknowledged partner. Trends Neurosci..

[bib2] Belu A., Schnitker J., Bertazzo S., Neumann E., Mayer D., Offenhäusser A., Santoro F. (2016). Ultra-thin resin embedding method for scanning electron microscopy of individual cells on high and low aspect ratio 3D nanostructures. J. Microsc..

[bib31] Bernardinelli Y., Nikonenko I., Muller D. (2014). Structural plasticity: mechanisms and contribution to developmental psychiatric disorders. Front. Neuroanat..

[bib3] Bishop D., Nikić I., Brinkoetter M., Knecht S., Potz S., Kerschensteiner M., Misgeld T. (2011). Near-infrared branding efficiently correlates light and electron microscopy. Nat. Methods.

[bib4] Blazquez-Llorca L., Hummel E., Zimmerman H., Zou C., Burgold S., Rietdorf J., Herms J. (2015). Correlation of two-photon *in vivo* imaging and FIB/SEM microscopy. J. Microsc..

[bib5] Bourne J.N., Harris K.M. (2008). Balancing structure and function at hippocampal dendritic spines. Annu. Rev. Neurosci..

[bib6] de Boer P., Hoogenboom J.P., Giepmans B.N.G. (2015). Correlated light and electron microscopy: ultrastructure lights up!. Nat. Methods.

[bib7] Feng G., Mellor R.H., Bernstein M., Keller-Peck C., Nguyen Q.T., Wallace M., Nerbonne J.M., Lichtman J.W., Sanes J.R. (2000). Imaging neuronal subsets in transgenic mice expressing multiple spectral variants of GFP. Neuron.

[bib8] Genoud C., Quairiaux C., Steiner P., Hirling H., Welker E., Knott G.W. (2006). Plasticity of astrocytic coverage and glutamate transporter expression in adult mouse cortex. PLoS Biol..

[bib9] Grabenbauer M. (2012). Correlative light and electron microscopy of GFP. Methods Cell Biol..

[bib10] Grabenbauer M., Geerts W.J.C., Fernadez-Rodriguez J., Hoenger A., Koster A.J., Nilsson T. (2005). Correlative microscopy and electron tomography of GFP through photooxidation. Nat. Methods.

[bib11] Heller J.P., Rusakov D.A. (2015). Morphological plasticity of astroglia: understanding synaptic microenvironment. Glia.

[bib12] Jung C.K.E., Herms J. (2014). Structural dynamics of dendritic spines are influenced by an environmental enrichment: an in vivo imaging study. Cereb. Cortex.

[bib13] Karreman M.A., Hyenne V., Schwab Y., Goetz J.G. (2016). Intravital correlative microscopy: imaging life at the nanoscale. Trends Cell Biol..

[bib14] Karreman M.A., Mercier L., Schieber N.L., Solecki G., Allio G., Winkler F., Ruthensteiner B., Goetz J.G., Schwab Y. (2016). Fast and precise targeting of single tumor cells in vivo by multimodal correlative microscopy. J. Cell Sci..

[bib32] Karreman M.A., Mercier L., Schieber N.L., Shibue T., Schwab Y., Goetz J.G. (2014). Correlating intravital multi-photon microscopy to 3D electron microscopy of invading tumor cells using anatomical reference points. PLoS One.

[bib15] Kizilyaprak C., Bittermann A.G., Daraspe J., Humbel B.M. (2014). FIB-SEM tomography in biology. Methods Mol. Biol..

[bib16] Knott G.W., Holtmaat A., Trachtenberg J.T., Svoboda K., Welker E. (2009). A protocol for preparing GFP-labeled neurons previously imaged in vivo and in slice preparations for light and electron microscopic analysis. Nat. Protoc..

[bib17] Kolotuev I., Bumbarger D.J., Labouesse M., Schwab Y. (2012). Targeted ultramicrotomy: a valuable tool for correlated light and electron microscopy of small model organisms. Methods Cell Biol..

[bib18] Kukulski W., Schorb M., Welsch S., Picco A., Kaksonen M., Briggs J.A.G., Müller-Reichert T., Verkade P. (2012). Precise, correlated fluorescence microscopy and electron tomography of lowicryl sections using fluorescent fiducial markers. Methods in Cell Biology.

[bib19] Li J., Erisir A., Cline H. (2011). In vivo time-lapse imaging and serial section electron microscopy reveal developmental synaptic rearrangements. Neuron.

[bib20] Lucas M.S., Günthert M., Bittermann A.G., de Marco A., Wepf R. (2017). Correlation of live-cell imaging with volume scanning electron microscopy. Methods Cell Biol..

[bib21] Luckner M., Wanner G. (2018). Precise and economic FIB/SEM for CLEM: with 2 nm voxels through mitosis. Histochem. Cell Biol..

[bib22] Maco B., Holtmaat A., Cantoni M., Kreshuk A., Straehle C.N., Hamprecht F.A., Knott G.W. (2013). Correlative in vivo 2 photon and focused ion beam scanning electron microscopy of cortical neurons. PLoS One.

[bib23] Maco B., Holtmaat A., Jorstad A., Fua P., Knott G.W., Müller-Reichert T., Verkade P. (2014). Correlative in vivo 2-photon imaging and focused ion beam scanning electron microscopy. Methods in Cell Biology.

[bib24] Masich S., Östberg T., Norlén L., Shupliakov O., Daneholt B. (2006). A procedure to deposit fiducial markers on vitreous cryo-sections for cellular tomography. J. Struct. Biol..

[bib25] Mironov A.A., Beznoussenko G.V. (2009). Correlative microscopy: a potent tool for the study of rare or unique cellular and tissue events. J. Microsc..

[bib33] Ostroff L.E., Manzur M.K., Cain C.K., LeDoux J.E. (2014). Synapses lacking astrocyte appear in the amygdala during consolidation of Pavlovian threat conditioning. J. Comp. Neurol..

[bib26] Panatier A., Arizono M., Nagerl U.V. (2014). Dissecting tripartite synapses with STED microscopy. Philos. Trans. R. Soc. Lond. B Biol. Sci..

[bib27] Schain A.J., Hill R.A., Grutzendler J. (2014). Label-free in vivo imaging of myelinated axons in health and disease with spectral confocal reflectance microscopy. Nat. Med..

[bib28] Schieber N.L., Machado P., Markert S.M., Stigloher C., Schwab Y., Steyer A.M. (2017). Minimal resin embedding of multicellular specimens for targeted FIB-SEM imaging. Methods Cell Biol..

[bib29] Sonomura T., Furuta T., Nakatani I., Yamamoto Y., Unzai T., Matsuda W., Iwai H., Yamanaka A., Uemura M., Kaneko T. (2013). Correlative analysis of immunoreactivity in confocal laser-scanning microscopy and scanning electron microscopy with focused ion beam milling. Front. Neural Circuits.

[bib30] Witcher M.R., Kirov S.A., Harris K.M. (2007). Plasticity of perisynaptic astroglia during synaptogenesis in the mature rat hippocampus. Glia.

