# Supplementary material for: Label-free 3D-CLEM Using Endogenous Tissue Landmarks
Source: iScience. 2018 Jul 20;6:92–101. doi: 10.1016/j.isci.2018.07.012 (PMC6137285; doi:10.1016/j.isci.2018.07.012)
Supplement: Document S1. Transparent Methods and Figures S1–S4 [file mmc1.pdf]

ISCI, Volume *6*

Supplemental Information

Label-free 3D-CLEM

Using Endogenous Tissue Landmarks

Manja Luckner, Steffen Burgold, Severin Filser, Maximilian Scheungrab, Yilmaz Niyaz, Eric Hummel, Gerhard Wanner, and Jochen Herms

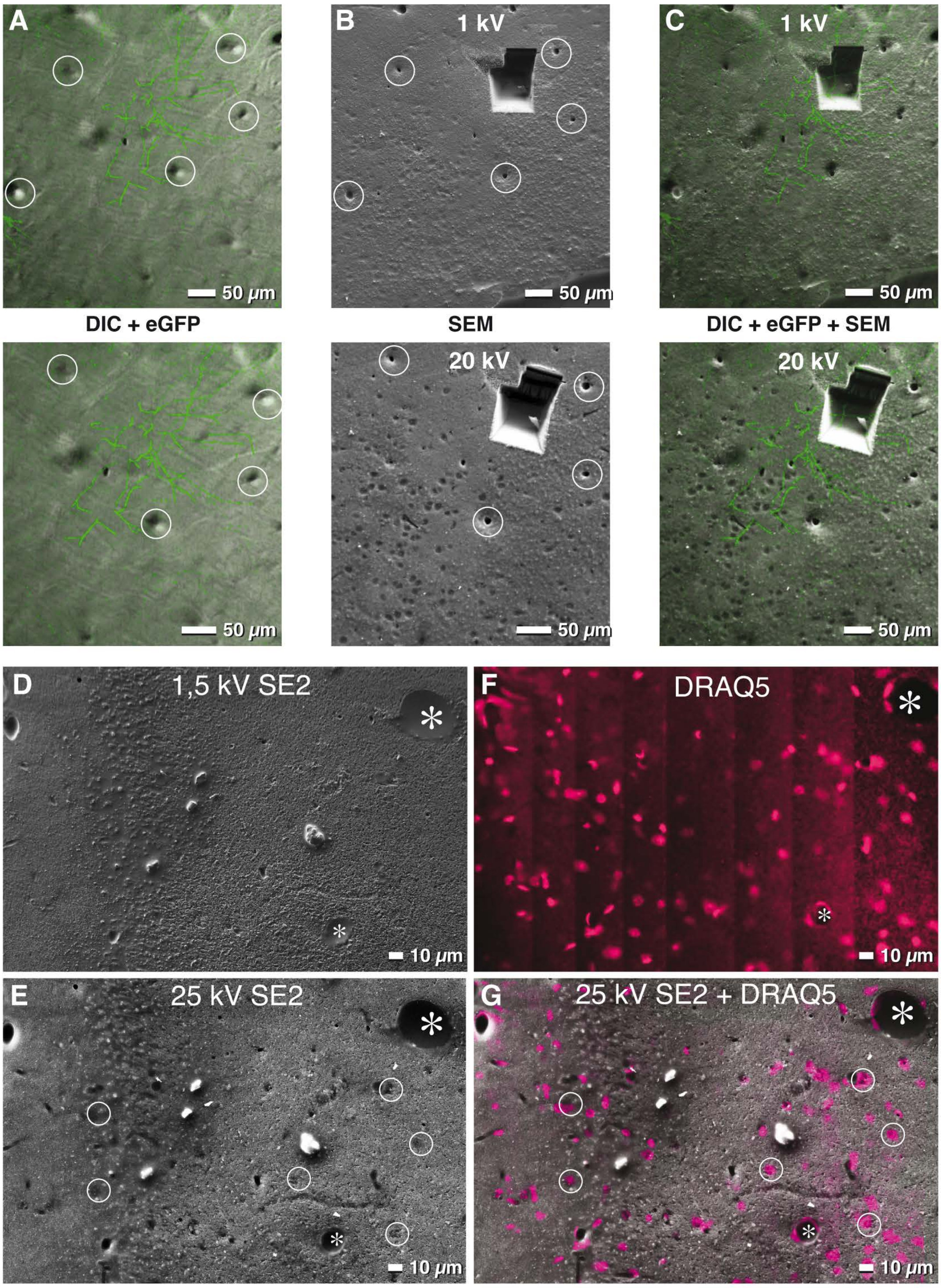

Figure S1. Vibratome sections at different kV, Related to Figure 1

(A) DIC images taken from the surface of the vibratome section and merged with a projection view of the dendrites (green = eGFP).

(B) SEM micrographs at different kV. Topographic and material contrast change are enhanced with increase of the accelerating voltage: Blood vessels are easily recognized as little holes (circles) at low and high kV. Sectioned nuclei with almost no topographic contrast at 1 kV, show a strong material contrast at 20 kV.

(C) Superimpostion of a DIC stack and an eGFP stack allows a precise localization of the target dendrite(s) in a range of a few µm.

(D-E) SEM micrographs at different kV. Different high voltages can be chosen to enhance either the topographic or material contrast of nuclei (circles): 1,5 kV (D) and 25 kV (E).

(F) From the DRAQ5 stack, 7 segments of different height in the stack were combined into a stitched image, representing only surface near nuclei.

(G) Superposition of micrographs enables precise correlation of nuclei (circles) and blood vessels (asterisk) in both, SEM an LSM.

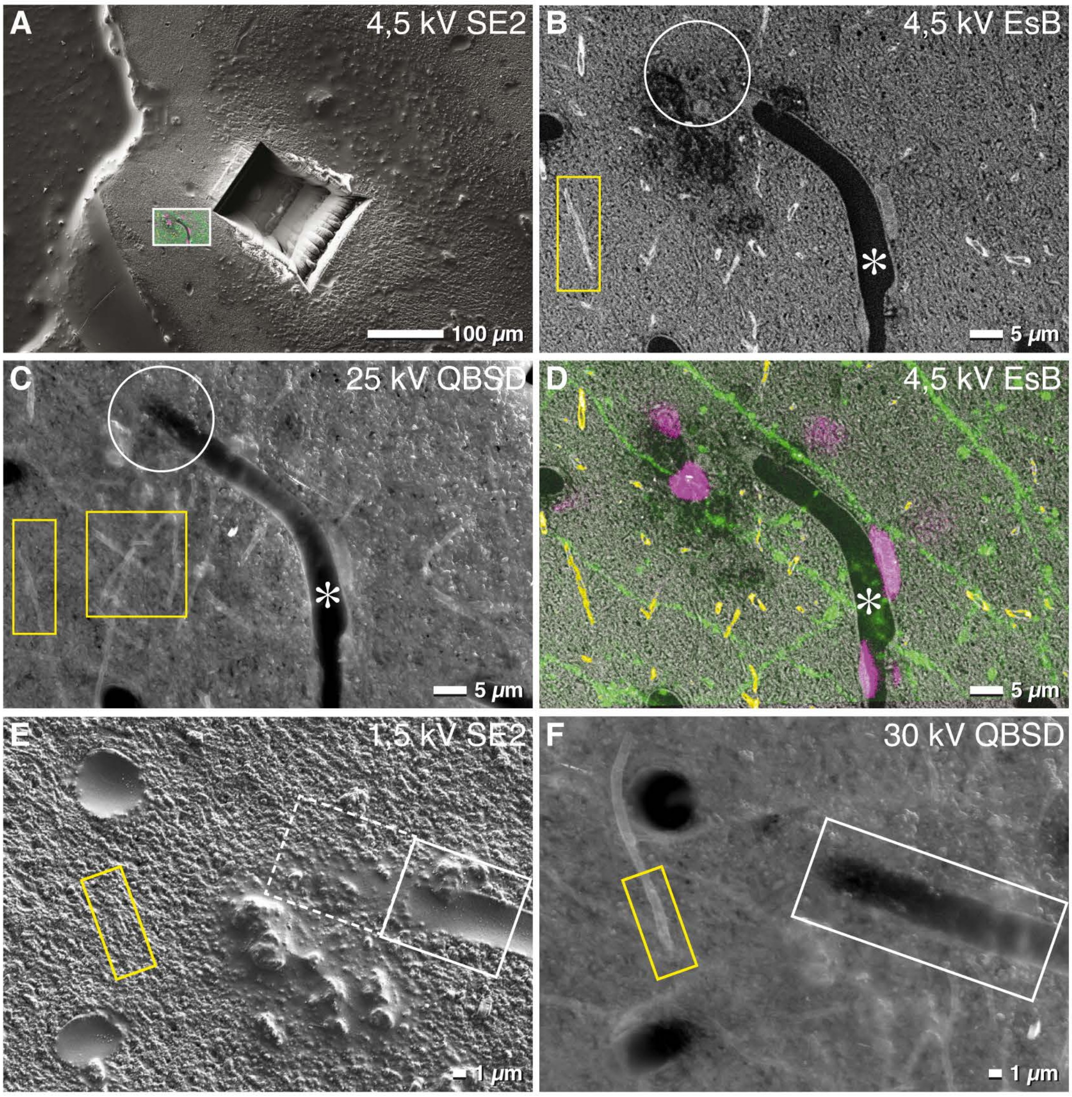

Figure S2. Myelinated axons in SEM, Related to Figure 1 and 2

(A) SEM micrograph of a vibratome slice. The trench indicates a FIB/SEM recorded target volume. Correlation with LM micrograph exhibits the next traget area.

(B-C) Myelinated axons are clearly visible in both, the EsB image at moderate kV (B) or in the QBSD image at high kV (C) due to their strong staining with osmium.

(D) Overlay of myelinated axons (yellow), nuclei (magenta) and dendrites (green) confirm the precision of surface correlation of CLSM and SEM. Blood vessels display at higher voltages a dark contrast (asterisk) or are visible at low kV by topographic contrast of the SE2 image (A).

(E-F) Comparison of 1,5 kV SE2 image (framed areas) with the QBSD image demonstrates the depth information given at high kV. Myelinated axons are clearly visible at high kV (F = yellow frame) and undetectable in the SE2-image (E = yellow frame).

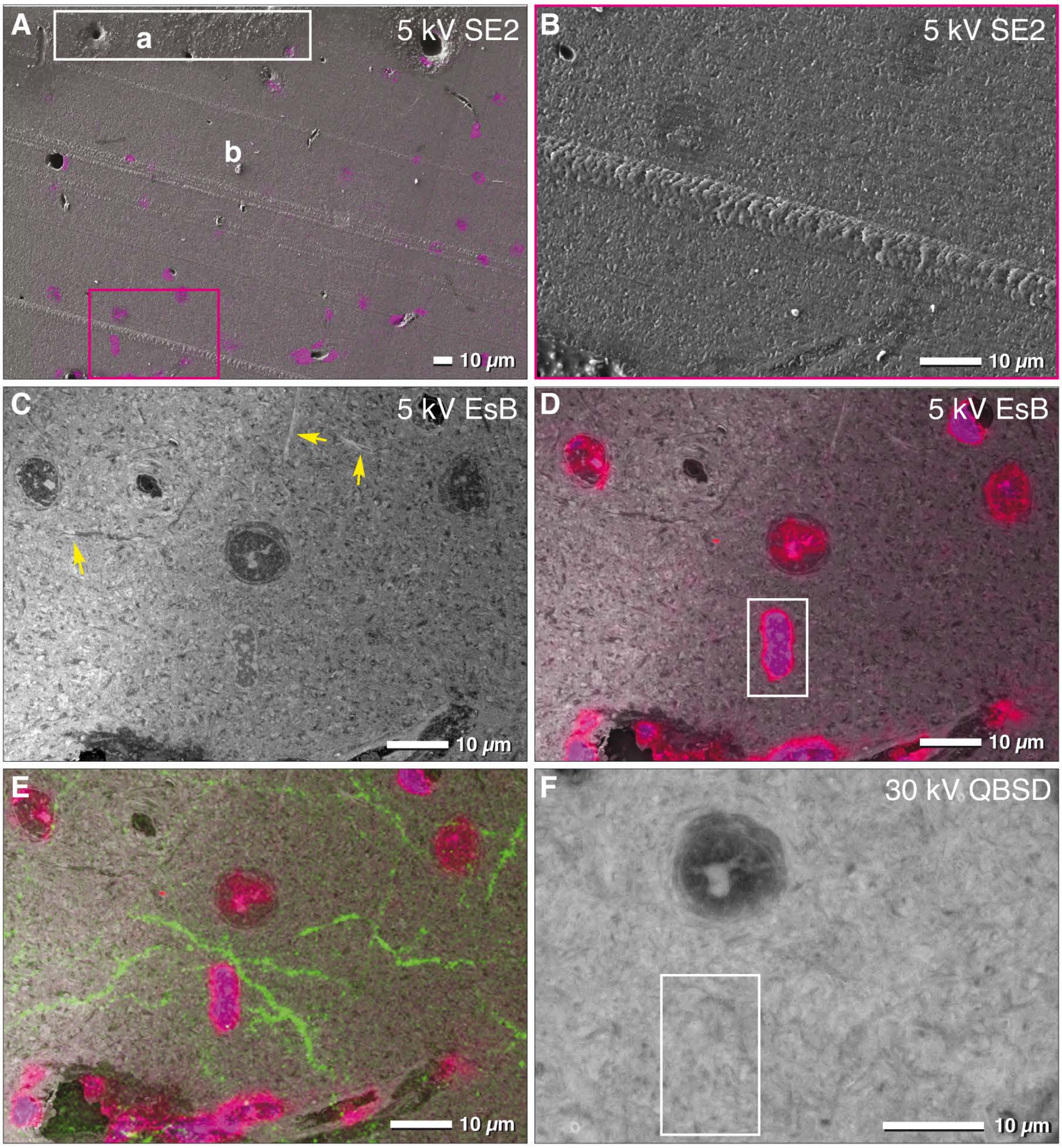

Figure S3. Enhancement of the material contrast after smoothing of vibratome sections, Related to Figure 1 and 2

(A) Correlation of light micrographs of stained nuclei (DRAQ5; LSM) with SEM micrographs of an embedded mouse brain section. For high resolution correlation, the specimen surface was smoothed by removal of few µm with a glass knife equipped ultra microtome. The difference in surface topography can be compared before (a = framed area; white) and after smoothing (b).

(B-E) At high magnification (framed area of A; magenta) nuclear details (nuclear envelope, heterochromatine) can be hardly recognized in the SE2 image (B) but they are clearly visible with the EsB-signal (C and D 5; kV). Axons (arrows) can be identified by their myelin sheets which give a high contrast due to the strong osmium staining. Superposition with the LM image of the nuclei (magenta) and dendrites (green) enable a precise correlation in the sub-micrometer range (E).

(F) The depth information is increased with high accelerating voltage (30 kV). Contrast of tangential sectioned nuclei becomes reduced (framed area; compare with framed area in D).

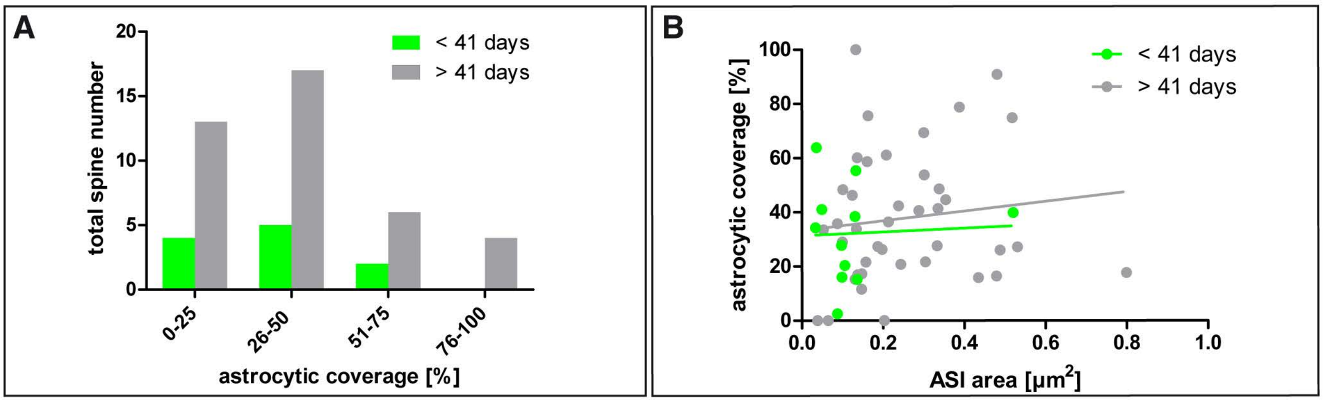

Figure S4. Astrocytes equally cover the synaptic perimeter of young and old spines, Related to Figure 4
(A) Distribution graphs of young (≤ 41 days) and old (≥ 41 days) dendritic spines according to astrocytic coverage of the synaptic perimeter.
(B) Correlation between astrocytic coverage and ASI area of young (≤ 41 days) and old (≥ 41 days) dendritic spines.

Transparent Methods

Animals

Female and male 2-3 months old heterozygous GFP-M mice (Tg(Thy1-EGFP)MJrs from Jackson Laboratory, Bar Harbor, Maine) were used (Feng et al., 2000). Mice were group-housed under pathogen-free conditions and bred in the animal housing facility of the Center of Stroke and Dementia Research, with food and water provided ad libitum (21 ± 1°C, at 12/12 hour light/dark cycle). All experiments were carried out in compliance with the National Guidelines for Animal Protection, Germany with the approval of the regional Animal care committee of the Government of Upper Bavaria, and were overseen by a veterinarian.

Cranial window implantation

Before use, surgical tools were sterilized in a glass-bead sterilizer (FST). Mice were anesthetized by an intra-peritoneal injection of ketamine/xylazine (140/10 mg/kg body weight, WDT, Bayer Health Care). In order to prevent cerebral edema Dexamethasone (6 mg/kg body weight, Sigma Aldrich) was injected intraperitoneally. Subsequently, mice were placed onto a heating blanket (37 °C) and the head was fixed in a stereotactic frame. Eyes were protected from drying by applying eye-ointment (Bepanthen, Bayer). The scalp was washed with swabs soaked with 70 % ethanol. A flap of skin covering the cranium was excised using small scissors. The periosteum was scraped away with a scalpel. The prospective craniotomy location (1.5-2.5 mm AP and 0-4 mm ML relative to bregma) was marked with a biopsy punch (diameter 4 mm, Integra LifeSciences). The exposed skull around the area of interest was covered with a thin layer of dental acrylic (iBond Self Etch, Hereaus Kulzer) and hardened with a LED polymerization lamp (Demi Plus, Kerr). A dental drill (Schick Technikmaster C1, Pluradent) was used to thin the skull around the marked area. After applying a drop of sterile phosphate buffered saline (DPBS, Gibco, Life Technologies) on the craniotomy the detached circular bone flap was removed with forceps. A circular coverslip (4 mm diameter, VWR International) was placed onto the craniotomy and glued to the skull with histoacryl adhesive (Aesculap). The exposed skull was covered with dental acrylic (Tetric Evoflow A1 Fill, Ivoclar Vivadent) and a head-post was attached parallel to the window for head-fixing mice in subsequent imaging sessions. After surgery, mice received a subcutaneous dose of the analgesic Carprophen (7.5 mg/kg body weight, Rimadyl, Pfizer) and antibiotic Cefotaxim (5

mg/kg body weight, Pharmore). Finally, mice were allowed to recover from surgery on a heating blanket.

Chronic *in vivo* two-photon microscopy

In vivo two-photon imaging started 4 weeks after cranial window implantation, using a multiphoton LSM 7 MP microscope (Zeiss) equipped with a fs-laser (Mai Tai DeepSee, Spectra-Physics), a 20x water immersion objective (W Plan-Apochromat 20x/1.0 NA, Zeiss) and a motorized stage. eGFP was excited at 920 nm and the emission was collected after a band pass filter from 470-550 nm by a non-descanned detector (photomultiplier tube GaAsP, Zeiss) directly after the objective. Throughout the imaging sessions, mice were anesthetized with isoflurane (1 % in oxygen, 0.5 l/min) and placed on a heating pad to keep body temperature at 37 °C (Fine Science Tools GmbH). A magnified image of the superficial cerebral blood vessels was acquired by using the camera port of the 7 MP microscope. The same ROIs were repositioned over time by alignment of the field of view based on the vascular pattern. Apical dendritic tufts of layer V pyramidal neurons in the somatosensory cortex were imaged in consecutive sessions at specified time points. For overview images, 3D stacks of 300 µm depth with 3 µm axial resolution and 1024×1024 pixels per image frame (0.4 µm/pixel) were acquired in multiphoton mode of the microscope. To resolve dendritic spines, high-resolution images from single dendrites were taken with 1 µm axial resolution and 512 × 256 pixels per image frame (0.1 µm per pixel). Individual imaging sessions lasted for no longer than 60 min with the laser power kept below 50 mW to avoid phototoxicity. Special care was taken to ensure consistent fluorescence levels both in space and time. After the fourth imaging session mice were placed into an enriched environment accordingly to a recently published protocol to enhance the structural plasticity of dendritic spines (Jung and Herms, 2014).

Preparation of brain sections

Mice were transcardially perfused with a mixture of formaldehyde and glutardialdehyde (Electron Microscopy Sciences, EMS) in PBS. A mixture of 0.5 % glutardialdehyde and 3.5 % formaldehyde was used in order to reduce background fluorescence from glutardialdehyde while preserving eGFP fluorescence and specimen ultrastructure. After 1 h perfusion mice were decapitated and the right

parietal bone with the ipsilateral window was removed. Subsequently, the mouse head was attached via head post to the stage of a vibratome (VT1000S, Leica Biosystems) to cut the brain tissue parallel to the imaging plane. This way, a single brain slice containing all cortical layers and the complete somato-sensory cortex was obtained. The cortical brain slice was incubated in 4% formaldehyde in PBS overnight at 4 °C, washed with PBS at the next day and subsequently stored in PBS at 4 °C. The fixed brain slice was further cut into 50 µm sections on a vibratome. Care was taken to keep the order of all obtained slices from top to bottom as well as not to flip them. Each slice was mounted onto a Cell-Tak™ (Corning®, Thermo Fisher Scientific) coated microscope slide (Superfrost, Menzel) with an adhesive, circular imaging spacer (Secure-Seal™, Grace Bio-Labs). The well of the imaging spacer was filled with 20 µM DRAQ5 (Biostatus) solution, sealed with a coverslip (No. 1.5, Zeiss) and incubated overnight at 4 °C.

LM microscopy

Slides were imaged in “tile scan” mode of an Apotome.2 wide field microscope (Zeiss) equipped with an EC Plan-NEOFLUAR 10x / 0.3 NA objective (Zeiss) to identify brain slices with the desired regions of interest. For bright field microscopy, the illumination aperture (NA 0.1) of the condenser was closed to increase image contrast. Emission of eGFP and DRAQ5 was collected using standard filter sets for green (490-606 nm) and far red (643-752 nm) (Zeiss). Confocal imaging was used (LSM 880 with AiryScan, Zeiss) to map all natural landmarks in 3D. Overview images of the region of interest with 3 µm axial resolution and 1024 × 1024 pixels per image frame (0.4 µm/pixel) were acquired with a Plan-Apochromat 20x / 0.8 NA air objective. High resolution image stacks (135 x 135 x 50 μm^3 with 0.13 µm in xy and 1 µm in z per pixel) of dendrites were acquired with a Plan-Apochromat 63x / 1.4 oil objective (Zeiss). DRAQ5 and eGFP were excited at 633 nm and 488 nm, respectively. Emission was collected from 500-560 nm (eGFP) and 650-700 nm (DRAQ5). Additionally, differential interference contrast images were acquired in the transmitted light channel. Furthermore, spectral confocal reflectance microscopy was used to visualize myelinated axons within the same volume. Axons were imaged with laser lines of 458 nm, 514 nm and 633 nm in a single track. The reflection mode of the microscope was used in combination with a partial mirror (80 % transmission, 20

% reflection) as main beam splitter. Images of all three channels were combined by using the average function of the Zen software (Version 2.3, Zeiss).

EM preparation

After light microscopic inspection mounted vibratome sections were uncovered by removal of the coverslip. Subsequently, samples were washed with cacodylate buffer (75 mM cacodylate 75 mM NaCl, 2 mM $MgCl_2$) and again fixed with 2,5 % glutardialdehyde in cacodylate buffer for 15 min, followed by 3 washing steps in cacodylate buffer. The tissue was post-fixed with 1 % OsO_4 and 1 % $K_4Fe(CN)_6$ in cacodylate buffer for 30 min, washed 3 times in ddH_2O, incubated with 1 % thiocarbohydrazide in ddH_2O for 30 minutes, washed with ddH_2O 3 times, followed by a second post-fixation with 1 % OsO_4 in ddH_2O for 30 min. Samples were further rinsed 3 times with ddH_2O, dehydrated in a graded series of acetone and incubated in 1 % uranyl acetate with 20 % acetone for 30 min. Subsequently, sections were embedded on the glass slide and infiltrated with 1:1 Hard-Plus Resin-812 in acetone for 10 minutes, 2:1 Hard-Plus Resin-812 in acetone for 30 min and finally in 100 % Hard-Plus Resin-812 for 1h at RT. Excessive resin was removed by centrifugation: the slide was placed in a 50 ml falcon tube with a tissue paper at the bottom and centrifuged at 1000 rpm for 2 min. After samples were polymerized for 3 days at 60 °C, the glass was trimmed with a diamond pen and mounted on an aluminum stub with colloidal silver. Finally, the glass was covered with colloidal silver and the entire sample was carbon coated (thickness of 15-20 nm) by evaporation.

FIB/SEM microscopy

Mouse brain tissues were imaged in an Auriga 40 FIB/SEM workstation (Carl Zeiss Microscopy GmbH) operating under SmartSEM (Carl Zeiss Microscopy GmbH) and Atlas3D software (Fibics Inc., Ottawa, Ontario, Canada). To facilitate correlation, the 50 µm thick brain slice was milled down to the surface of the glass slide, which was used as a reference. Subsequently, a 70 µm long ramp was milled by FIB (for vibratome sections with a thickness of 50 µm and a FIB tilt angle of 54°, the length of the milling ramp can be calculated as tan54° = 1.374. In this case, the trapezoid trench has to be approx. 70 µm long). After milling of 20 µm in z-direction milling was stopped, the ion beam current reduced to 10 nA, and the milling depth increased to

10-20 µm. After additional 20 µm of length, the trench was milled with a depth of 30 µm, until the beam reaches the glass slide. While milling the ramp to get access to the target volume, key frames were taken to inspect the correlation landmarks (blood vessels, nuclei and myelinated axons) to determine the position of the target dendrite in all three dimensions. Final milling parameters were set to 1-2 nA milling current of the Ga-emitter. With each cycle 10-15 nm of epoxy resin was removed. SEM images were recorded with an aperture of 60 µm in the high current mode at 1.5 kV of the in-lens EsB detector with the EsB grid set to -800-1200 V. Key frames were imaged with a pixel size of 20-27 nm and the ROI with a pixel size of 5-8 nm. Images series of 3000-4000 sections were recorded. In the synchronous mode of the ATLAS-System, the milling current and depth were adjusted to match milling time with exposure time set to 1 min. Automatic correction of focus (*auto tune*) and astigmatism (*auto stig*) was applied every 30 minutes.

3D reconstruction of LM and FIB/SEM image stacks

LM images of blood vessels, nuclei and myelinated axons were reconstructed in 3D using Imaris (Version 7.7.2, Bitplane). FIB/SEM image stacks were aligned, segmented and 3D reconstructed in Amira (FEI Company). For registration of the LM and FIB/SEM datasets, both 3D reconstructions were fit into each other manually by rotation and translation. FIB/SEM volume was used as a reference to adjust the LM/DIC reconstruction. Registration was performed by successive alignation of blood vessels, nuclei and finally myelinated axons to increase the precision of the correlation.

Image analysis

Amira 3D reconstructions of dendritic spines, corresponding presynapses and perisynaptic astrocytic processes were imported in Blender software for morphometric analysis. Volumes, surfaces and axon spine interfaces (ASI) were quantified with the plugins “NeuroMorph Measurement Tools” and “NeuroMorph Proximity Analysis” (Jorstad, A., Nigro, B., Cali, C. *et al.* 2015 and Barnes *et al.* 2015). Astrocytic coverage was quantified by measuring the total perimeter of the ASI and the proportion that was covered by the astrocyte. To calculate distance distributions of endogenous landmarks in GraphPad Prism 5, distances between 10 randomly chosen dendrites and their closest natural landmarks were measured and

quantified with the 3D measurement tool in Imaris (Version 7.7.2, Bitplane). Dendritic spine lifetime was determined manually off-line by analyzing the corresponding 2-photon micrograph time series. Dendritic spines were defined as stable if their locations did not change along the dendritic shaft between consecutive imaging sessions (acceptable range < 1 µm). Spines, which emerged or disappeared over two consecutive imaging sessions, were assigned as newly gained or lost, respectively. Resolution limitations in the z-plane restricted our analysis to laterally protruding spines. For illustration purpose only, image stacks were deconvolved (AutoQuantX2, Media Cybernetics) and adjusted for contrast and brightness. Figures were created in Adobe Photoshop/Illustrator CS6 and supplementary movies were made in Amira (Thermo Fisher Scientific™).

Key Resource Table

REAGENT or RESOURCE	SOURCE	IDENTIFIER
**Chemicals, Peptides, and Recombinant Proteins**		
20% Formaldehyde	SCIENCE SERVICES	Cat# E15713
25% Glutardialdehyde	SCIENCE SERVICES	Cat# E16216
Cell-Tak™	Thermo Fisher Scientific	Cat# 10317081
DAPI	Thermo Fisher Scientific	Cat# D1306
DRAQ5	Biostatus	Cat# DR50050
Fetal Bovine Serum (FBS)	GIBCO	Cat# 10270-106
Osmium Tetroxide	SCIENCE SERVICES	Cat# E19130
PBS	GIBCO	Cat# 20012-068
Potassium hexacyanoferrate(II) trihydrate	Sigma-Aldrich	Cat# 455946
Sodium cacodylate trihydrate	MERCK	Cat# 20840-100G-F
Thiocarbohydrazide	Sigma-Aldrich	Cat# 223220
**Experimental Models: Organisms/Strains**		
Mouse: STOCK Tg(Thy1-EGFP)MJrs/J	The Jackson Laboratory	Stock# 007788
**Software and Algorithms**		
Adobe Photoshop CC	Adobe Systems Software Ireland Limited	http://www.adobe.com
Altlas3D	Fibics Inc.	
Amira 6.2	Thermo Fisher Scientific	https://www.fei.com/software/amira-3d-for-life-sciences/

Blender<br>Plugin:<br>NeuroMorph Measurement Tools<br>NeuroMorph Proximity Analysis	The Blender Foundation	https://www.blender.org/
GraphPad Prism	GraphPad Software Inc,	http://www.graphpad.com/ scientific-software/prism/
ImageJ (1.50, Java 1.8.0_60, 64 bit)	NIH	https://imagej.nih.gov/ij/; RRID:SCR_003070
Imaris 7.7.2	Bitplane	http://www.bitplane.com/
SmartSEM	Carl Zeiss Microscopy GmbH	N/A
Zen 2.3	Carl Zeiss Microscopy GmbH	N/A
